# B-type brain natriuretic peptide as a measure of the severity of hand-foot-mouth disease: a case-control study

**DOI:** 10.1186/s12879-017-2734-9

**Published:** 2017-09-29

**Authors:** AYuan Zhang, Lin Yang, Pengfei Guo, Shaojun Li, Xiaoxiao Ao, Feng Xu, Liping Tan

**Affiliations:** 1 Department of Emergency, Children’s Hospital of Chongqing Medical University, No. 136, Zhongshan 2 Road, Yuzhong district, Chongqing, 400014 China; 2 Department of Respiratory Medicine, Sichuan Provincial Hospital For Women & Children, Chongqing, China; 3 Pediatric Intensive Care Unit, Children’s Hospital of Chongqing Medical University, Chongqing, China; 4 Ministry of Education Key Laboratory of Child Development and Disorders, Chongqing Key Laboratory of Pediatrics, China International Science and Technology Cooperation Base of Child Development and Critical Disorders, Chongqing, China

**Keywords:** HFMD, BNP, Diagnostic indicator, Cardiopulmonary complications

## Abstract

**Background:**

Hand-foot-mouth disease (HFMD) is an acute infectious disease caused by enteroviruses, and HFMD complicated by cardiopulmonary failure has a high mortality. B type natriuretic peptide (BNP) is widely applied in monitoring cardiovascular disorders, and thus, we investigated whether this index was associated with the severity of HFMD and the outcome in severe HFMD.

**Methods:**

Serum BNP, lactate, and glucose levels as well as white blood cell (WBC) count, PaO_2_/FiO_2_, and cardiac output (CO) were analyzed in the 83 enrolled HFMD patients according to different conditions (common, severe, and critical; with and without complication; and survivors and non-survivors). The control group consisted of 29 patients with respiratory tract infections.

**Results:**

No significant differences in CO were observed between the groups. Serum lactate, glucose, BNP, and WBC levels in the critical group were significantly higher than those in the severe, common, and control groups (*p* < 0.01 or 0.05). The PaO_2_/FiO_2_ ratio was significantly lower in the critical group (214.286 ± 154.346) than in the other groups. According to logistic regression analysis, the areas under the curve for serum BNP, glucose, and PaO_2_/FiO_2_ of the patients with complications were 0.774, 0.738, and 0.75, respectively. Moreover, the BNP level was significantly higher in HFMD patients with complications and non-survivors.

**Conclusion:**

Our findings indicate that BNP could be a biochemical indicator for severe (critical) HFMD and used for prognosis in terms of complications and death. Combined with Glu and PaO_2_/FiO_2_ and clinical symptoms of HFMD, the value of BNP as an indicator became more precise and specific. Our results may provide another valuable, objective biochemical indicator for severe HFMD.

**Trial registration number:**

ChiCTR-DDT-14004576. Name of registry: Chinese Clinical Trial Registry. Date of registration: 2014–09-21.

## Background

Hand-foot-mouth disease (HFMD) is an acute infectious disease caused by various enteroviruses, and the most common pathogens are Coxsackievirus A16 (CA16) and Enterovirus 71 (EV71). Preschool children are easily infected, and the clinical characteristics of ordinary HFMD are fever along with flat discolored spots and bumps on the hands, feet, and mouth. In severe cases of HFMD, varying degrees of neurological complications (such as encephalitis and neurogenic pulmonary edema) and circulatory complications can occur. This severe form of HFMD has a high mortality rate, and survivors often suffer from long-term sequelae [1, 2].

HFMD is an epidemic disease in most parts of the world, and the incidence of HFMD has remained high in recent years, presenting a bimodal distribution status over the course of the year [3, 4]. According to the 2011 version of the Expert Consensus of Clinical Treatment in Severe Cases of Enterovirus 71 Infection in China [5], HFMD cases are classified into two types, common and severe, and the severe type is further divided into severe HFMD and critical HFMD. The clinical progression of HFMD is described in five clinical phases: foot and mouth eruption (phase I), nervous system involvement (phase II), early cardiopulmonary failure (phase III), cardiopulmonary failure (phase IV), and recovery (phase V). Most cases of death occur in patients who reach Phases III and IV, and thus, these two phases represent the crucial period for treating severe HFMD. However, the diagnosis and prognosis of HFMD mainly rely on the patients’ clinical manifestations and signs. Therefore, the identification of objective experimental and biochemical indicators would be valuable for the prevention and clinical treatment of HFMD. Because cardiopulmonary failure is the major cause of death among HFMD patients [6], the primary goal in treating severe HFMD is to maintain the stability of cardiopulmonary circulation function.

Given that B type natriuretic peptide (BNP) is widely applied in monitoring cardiovascular disorders [7–9], we hypothesized that introduction of this index into HFMD management could be helpful in the diagnosis and prognosis of severe HFMD. BNP is mainly secreted by the ventricular myocardium, which has many physiopathological functions, such as diuresis through the natriuretic process, blood vessel dilation, lowering peripheral vascular resistance, reducing the activity of rennin-angiotensin-aldosterone, and decreasing adrenaline and the endothelial response, thereby regulating the blood volume, blood pressure, and salt balance within the human body [10]. Once myocardial cells are subjected to stretching or the myocardial wall is exposed to increased pressure, the original BNP stored in myocardial cells is released quickly and broken down into active BNP and inactive NT-proBNP [11]. Numerous studies have demonstrated that BNP is an important and valuable biomarker in the diagnosis, prognosis, and risk evaluation of conditions such as acute and chronic heart failure, sepsis, and respiratory failure due to cardiac diseases [12].

To test our hypothesis in the present study, we analyzed the data of 83 HFMD patients with or without severe complications treated in our hospital from April 2014 to March 2015 according to the severity of HFMD (common, severe, and critical; with and without complications; and survivors and non-survivors). The clinical value of BNP in relation to the clinical symptoms of HFMD was estimated and discussed.

## Methods

### Patients

For this study, we enrolled 83 HFMD patients, including 44 boys and 39 girls, aged 0–7 years (mean, 26.76 ± 18.47 months) who were hospitalized in the Department of Infectious Diseases of the Children’s Hospital, Chongqing Medical University between April 1, 2014 and March 31, 2015. Patients with atypical spots and bumps, a complicating primary lung disease, or another viral encephalitis or meningitis were not included. For the control group, 29 patients (18 boys and 11 girls; mean age, 27.36 ± 14.95 months) with respiratory tract infections who were hospitalized in the Department of Pediatrics of the same hospital during approximately the same time period were enrolled. All guardians of the patients consented to patients’ participation in this study and signed a consent form. This study was authorized by the Institutional Human Ethics Committee of Children’s Hospital of Chongqing Medical University.

### HFMD diagnosis and classification

All 83 patients were diagnosed according to the Guidelines for Diagnosis and Treatment of HFMD (2010 edited version) by the Ministry of Health of the People's Republic of China (http://www.moh.gov.cn/mohyzs/s3586/201004/46884.shtml). Onset age; epidemic season; typical flat discolored spots and bumps on hands, feet, mouth, and buttocks; fever; and serological test [including EV-A71 *specific-IgM* (ELISA) or HFMD real-time PCR] were considered as major diagnostic references.

The 83 HFMD cases were divided into three groups according to the clinical characteristics: common, severe, and critical HFMD. Patients with only rashes on hands, feet, mouth, and buttocks with or without fever were included in the common group. Patients who also had symptoms of neurological complications including lethargy, vomiting, headache, myoclonus, limb tremor, ataxia, nystagmus, powerless or acute flaccid paralysis, and eye movement disorders were included in the severe group. Patients assigned to the critical group had one of the following conditions: coma, frequent convulsions, dyspnea, brain hernia, bloody foamy sputum, cyanosis, pulmonary rales, shock, or another form of circulatory failure.

### Classification according to HFMD stages

Patient groups were also created according to the stages of HFMD defined by the HFMD clinical treatment expert consensus (2011 edition) [5]: phase I HFMD, the rash period (typically included in the common group); phase II HFMD, nervous system symptoms (typically included in the severe group); phase III, early cardiorespiratory failure (typically included in the critical group); and phase IV, cardiopulmonary function failure (typically included in the critical group. None of the patients had phase V HFMD (recovery period) at the start of the study.

### Blood gas analysis

A 0.5-ml sample of arterial blood was collected from each patient into a heparin-containing tube for blood gas analysis. The blood gases, including arterial partial pressure of oxygen (PaO_2_), and fraction of inspired oxygen (FiO_2_), were analyzed using a GEM Premier 3000 blood gas analyzer (Instrumentation Laboratory, Bedford, MA, USA) according to the instrumental operation protocol.

### Serum lactate, glucose, and BNP measurement

A 2-ml sample of venous blood was collected from each patient into an EDTA coagulant-containing tube and kept in a 37 °C incubator for 2 h before serum (supernatant) collection for measurement of lactate (Lac), glucose (Gluc), and BNP levels as well as routine blood tests. BNP concentration was detected by a chemiluminescence method using the ADVIA Centaur CP Immunoassay System (SIEMENS, Munich. Germany).

### Cardiac function detection

The cardiac output (CO) of the patients was evaluated by noninvasive CO monitoring using USCOM 1A instruments (Australia) at the same time.

### Statistical analysis

All data were analyzed with SPSS 12.0 statistical software (http://spss.software.informer.com/12.0/) and are presented as mean *±* standard deviation. *p* < 0.05 indicated a statistically significant difference. Comparisons of normally distributed data were analyzed by analysis of variance (ANOVA) and using *Student–Newman–Keuls* (*SNK*) method. Comparisons of non-normally distributed data were analyzed with a nonparametric test (Kruskal-Wallis test for K-sample and Kolmorogov-Smirnov test for two-independent samples). Multiple factor analysis was performed via multivariate logistic regression for elimination of variables. The receiver operating characteristic (ROC) curve for BNP level was plotted to calculate the area under the curve (AUC). The Youden index was calculated according to the ROC curve [Youden index = the sensitivity (Se) + specific degrees (Sp) - 1]. The largest Youden index threshold indicated the best diagnostic value. Correlation analysis was carried out using Spearman’s rank correlation coefficient method, and the inspection level (*p*) was 0.05.

## Results

### Patient characteristics

Of the 83 enrolled HFMD patients, 30 were assigned to the common group (18 boys and 12 girls; mean age, 25.567 ± 14.474 months), 29 to the severe group (15 boys and 14 girls; mean age, 27.973 ± 13.677 months), and 24 to the critical group (11 boys and 13 girls; mean age, 29.375 ± 14.832 months). Twenty of the 24 patients in the critical group died during the study period (death rate, 83.33%). Age and gender did not differ significantly between the control and HFMD groups (*p* > 0.05).

### Differences in the blood gas and serum biochemical factors

The Lac and glucose as well as the WBC in the critical group were significantly higher than those in the severe, common, and control groups (*p* < 0.05; Table 1). The data for BNP were not normally distributed. The nonparametric analysis showed significant differences in BNP levels (γ^2^ 45.388, *p* < 0.05) among the groups. Nonparametric test was used for comparison between the two groups, and the *p* value was set at 0.01. Among these variables, the BNP level in the critical group (38.83 pmol/L; range, 15.85–114.38 pmol/L) presented the most significant difference (*p* < 0.01) from the levels in the other groups (2.15–3.42 pmol/L). The peak BNP concentration even reached as high as 343.99 pmol/L. The CO showed no significant difference among the groups. The PaO_2_/FiO_2_ ratio was significantly lower in the critical group (214.286 ± 154.346) than in the other groups (373.67–408.498; Table 1). Overall, these results indicated that a serum BNP concentration of 70 pmol/L could be an indicator of critical HFMD.

**Table 1:** Blood gas and serum biochemical factors in four HFMD groups ($\overline{x}\pm s$)

Index	Critical	Severe	Common	Control
n	24	29	30	29
Lac (mmol/L)	2.775 ± 1.535	1.459 ± 0.916^a^	1.498 ± 0.673^a^	1.345 ± 0.580^a^
Glu (mmol/L)	13.237 ± 6.936	6.479 ± 2.171^a^	5.843 ± 1.411^a^	5.324 ± 0.930^a^
PaO_2_/FiO_2_	214.286 ± 154.346	373.671 ± 98.993^a^	438.929 ± 81.367^a,c^	408.498 ± 28.911^ac^
BNP (pmol/L)	38.83 (15.85,114.38)	3.42 (1.84,6.95)^b^	2.15 (0.87,5.56)^b^	2.71 (1.53,6.18)^b^
CO (L/min)	2.433 ± 0.623	2.660 ± 0.621	2.735 ± 0.681	2.781 ± 0.640
WBC (×10^9^/L)	17.245 ± 6.022	10.111 ± 2.617^a^	10.697 ± 2.501^a^	9.352 ± 3.236^a^

^a^ *p* < 0.05, comparison of critical group to other groups

^b^ *p* < 0.01, comparison of critical group to other groups [the data for BNP were not normally distributed. The nonparametric analysis showed significant differences in BNP levels (γ^2^ 45.388, *p* < 0.05) among the groups. Nonparametric test was used for comparison between the two groups. *p* value was set as 0.01]

^c^ *p* < 0.05, comparison of severe group to other groups

### Blood gas and serum biochemical factors in different HFMD stages

The WBC count and CO index data of the four groups were normally distributed. ANOVA showed that the overall difference in WBC count was statistically significant. The WBC count of phase IV patients was significantly higher than those of the other three groups (*p* < 0.05). The WBC count of phase III patients also was significantly greater than those of phase I and phase II patients (*p* < 0.05). There was no significant difference in the WBC count between phase II and phase I patients (*p* > 0.05). The CO value for phase IV patients appeared to be lower than that in the other three groups, but there was no significant difference statistically between two groups overall (*p* > 0.05).

The data for BNP, Lac, and Glu levels as well as PaO_2_/FiO_2_ were not normally distributed. The nonparametric analysis showed significant differences in Lac (χ^2^ test: 21.907, *p* < 0.01), Glu (χ^2^ test: 36.193, *p* < 0.01), and BNP (χ^2^ test: 39.853, *p* < 0.01) levels as well as PaO_2_/FiO_2_ (χ^2^ test: 36.422, *p* < 0.01) among the groups. In comparisons between two groups, no differences in Lac, Glu, BNP, and oxygenation index (*p* > 0.05) were found between phase IV and phase III patients, but these indexes in phase IV patients were significantly higher than those in phase I and phase II patients (*p* > 0.01). In addition, these indexes in phase III patients were significantly higher than those in phase I and phase II patients (*p* < 0.01). No statistically significant differences were found in any indexes between phase I and phase II patients (all *p* > 0.05; Table 2).

**Table 2:** Blood gas and serum biochemical factors in four stages of HFMD ($\overline{x}$ *± s*)

Indexes	Phase I	Phase II	Phase III	Phase IV
n	30	29	13	11
Lac (mmol/L)	1.3 (1.0,1.93)	1.1 (1.0,1.6)	2.1 (1.55,3.05)	2.3 (1.9,4.3)
Glu (mmol/L)	5.8 (4.68,6.55)	5.99 (5.15,6.90)	11.2 (7.90,14.25)	12.2 (9.7,19.9)
PaO_2_/FiO_2_	453.57 (416.07,487.50)	421.43 (408.93,460.71)	228.75 (136.63,408.93)	135.0 (80.36,256.88)
BNP (pmol/L)	5.8 (4.68,6.55)	3.42 (1.84,6.95)	54.78 (16.74,123.74)	25.82 (8.48,44.83)
CO (L/min)	2.735 ± 0.681	2.660 ± 0.621	2.289 ± 0.477	2.692 ± 0.704
WBC(×10^9^/L)	10.697 ± 2.501^a,c^	10.111 ± 2.617^a,c^	15.183 ± 5.123^a^	19.682 ± 6.313^c^

^a^ *p* < 0.05, comparison of Phase IV group to other groups

^b^ *p* < 0.01, comparison of Phase IV group to other groups

^c^ *p* < 0.05, comparison of Phase III group to other groups

### Comparison of indexes between patients with and without complications

To determine the clinical value of the observed parameters among HFMD patients, we divided the patients into groups according to whether they experienced complications. Thus, we combined the data from the severe HFMD and critical HFMD groups, both of which had complication, for comparison to data from the common group (Table 3). The Lac, Glu, BNP, and WBC levels in patients with complications were significantly higher than those in patients without complications (*p* = 0.00–0.043). The PaO_2_/FiO_2_ ratio in patients with complications was significantly lower than that in common HFMD group (*p* = 0.00). The level of CO did not differ between the patients with and without complications (Table 3).

**Table 3:** Comparison of the patients with complications to patients without complications (± s)

Indexes	With complications	Without complications	t/z	*p*
n	53	30		
BNP (pmol/L)	8.48 (2.95,32.80)	2.15 (0.87,5.56)	−4.133	0.000
Lac (mmol/L)	2.055 ± 1.390	1.498 ± 0.673	2.056	0.043
WBC (×10^9/L)	13.342 ± 5.708	10.697 ± 2.501	2.406	0.018
Glu (mmol/L)	7 (5.75,11.80)	5.80 (4.68,6.55)	−3.593	0.000
CO (L/min)	2.557 ± 0.626	2.735 ± 0.681	−1.204	0.232
PaO_2_/FiO_2_ (mmHg)	403.57 (161.34,439.29)	453.57 (416.07,487.50)	−3.874	0.000

**Fig. 1:**
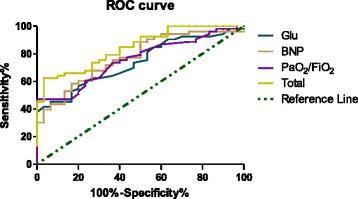
ROC curves for BNP, Glu, and PaO_2_/FiO_2_ in the logistic regression analysis comparing HFMD patients with and without complications

Next, the statistically significant variables from Table 3 were analyzed via a logistic regression model to select the meaningful variables (Glu, BNP, PaO_2_/FiO_2_; Table 4) to estimate whether they could predict the severity of HFMD (with complications). The corresponding ROC curves were prepared (Fig. 1; Table 5), and AUC values for BNP, Glu, and PaO_2_/FiO_2_ were 0.774, 0.738, and 0.75, respectively. All of these AUC values were greater than 0.5, indicating that the three indexes were valuable predictors for the severity of HFMD. These results suggest that a BNP concentration of ≥35 pmol/L (*p* < 0.01) could be an indicator of HFMD with complication.

**Table 4:** Logistic regression of BNP, Glu, and PaO_2_/FiO_2_ between complication and non-complication patients

Factors	B	SE	Wald	*p*	OR	95% CI
Constant term	3.509	2.379	2.176	0.140		
BNP	0.076	0.041	3.502	0.061	1.079	0.996–1.169
Glu	0.369	0.184	4.039	0.044	1.447	1.009–2.075
PaO_2_/FiO_2_	−0.014	0.006	6.044	0.014	0.986	0.975–0.997

*SE* Standard error, *OR* Odds ratio; 95% CI, 95% confidence interval

**Table 5:** ROC curve analysis of BNP, Glu, and PaO_2_/FiO_2_ between complication and non-complication patients

Indexes	AUC	*p*	95%CI	Youden
BNP	0.774	0.000	0.674–0.875	0.418
Glu	0.738	0.000	0.633–0.843	0.386
PaO_2_/FiO_2_	0.757	0.000	0.655–0.858	0.472
Total	0.845	0.000	0.764–0.926	0.589

### Comparison of indexes between survivors and non-survivors

In the critical HFMD group, 20 of 24 patients died due to serious complications. Toward to the goal of identifying indicators of severe HFMD, we analyzed the differences in the observed indexes between HFMD patients who died and those who survived. Serum Lac and Glu concentrations were significantly higher in non-survivors than in survivors, and PaO_2_/FiO_2_ was significantly lower in non-survivors compared with survivors. However, BNP level, WBC count, and CO did not differ significantly between the two groups (Tables 6 and 7).

**Table 6:** Comparison of indexes between survivors and non-survivors of critical HFMD

Index	Non-survivors	Survivors	t/z	*p*
n	4	20		
BNP (pmol/L)	41.08 (18.34,101.75)	38.83 (14.75,115.21)	−0.155	0.877
Lac (mmol/L)	2.35 (1.93,3.78)	1.65 (1.23,2.00)	−2.209	0.027
WBC (×10^9/L)	17.66 (12.83,22.84)	11.20 (9.92,19.28)	−1.704	0.088
Glu (mmol/L)	12.70 (10.63,15.20)	6.95 (6.23,9.55)	−2.557	0.011
CO (L/min)	2.379 ± 0.641	2.700 ± 0.504	- 0.939	0.358
PaO_2_/FiO_2_ (mmHg)	141.0 (113.30,242.81)	429.46 (315.98,644.20)	−2.324	0.020

**Table 7:** The multi-factor logistic regression for predicted death group

Factor	B	SE	Waldχ2	*p*	OR	95%CI
Constant term	4.661	1.707	7.459	0.006	0.979	0.979–0.999
PaO_2_/FiO_2_	−0.011	0.005	4.657	0.031		

## Discussion

As a viral-infection disease, HFMD has no specific treatment. Prevention, early diagnosis, respiratory support, and treatment of brain injury are the key measures to reducing the mortality of severe HFMD [5]. For prevention of HFMD, Zhu et al. performed phase III clinical trial studies with an EV71-inactivated vaccine in China [13, 14] and reported satisfactory results in terms of the protective effect and safety of the vaccine. The vaccine received a new drug certificate from the Chinese National Food Drug Administration on December 3, 2015.

For clinical treatment of severe HFMD, limiting the fluid volume and actively controlling intracranial pressure are recommended [5]; however, other opinions suggest the use of glucocorticoids and intravenous immunoglobulin [6]. Some scholars believe that early application of glucocorticoids provides an anti-inflammatory effect to reduce capillary permeability, effectively promoting recovery from pulmonary edema and brain edema [15]. Here, we studied potential indexes for the early diagnosis of severe HFMD and showed that the serum BNP level combined with other HFMD symptoms could be a biochemical indicator for complications (BNP >8.4 pmol/L), critical disease (BNP >38.8 pmol/L), and death (BNP >41.1 pmol/L) HFMD patients.

The pathological mechanism of HFMD has been a topic of research for decades. Some studies have focused on central nervous system complications in HFMD. Enterovirus was found to invade the central nervous system through lymphatic channels or peripheral sympathetic nerve channels located in the hypothalamus, dorsal nucleus, and the ventral brainstem, medial brainstem, and tail of the brainstem to cause hyperfunction of the sympathetic nervous system [16]. Pulmonary vascular permeability in severe HFMD has also been a research focus. The involvement of the central nervous system causes sympathetic nerve excitement, which leads to imbalance between beta adrenergic receptor and alpha adrenaline receptor levels in the lung, resulting in an increase in the proportion of alpha adrenaline receptor. These alterations directly increase blood capillary permeability in the lungs and plasma extravasation and cause pulmonary edema [17].

Another severe pathological effect in HFMD is immune inflammation. The complications of brain stem encephalitis and pulmonary edema caused by EV71 virus in HFMD patients are always accompanied by significant increases in serum interleukin (IL)-13, IL-10, IL-8, interferon gamma (IFN-γ), monocyte chemotactic factor 1 cytokine, natural killer (NK) cells, CD4^+^ T lymphocytes, and CD8^+^ T lymphocytes [18]. Chang et al. found that the human leukocyte antigen serotype (HLA) genes A33 and HLA-A2 are susceptibility genes [19]. The occurrence of neurogenic pulmonary edema is caused by the nervous, immune, and endocrine systems together, and as the result of comprehensive shaping by many factors, its mechanism is not fully clear.

BNP and natriuretic peptide receptors play roles in several physiological processes, such as diuresis, dilation of blood vessels, and inhibition of the rennin angiotensin-aldosterone system [10]. Recently, studies concerning BNP have greatly enriched the field of cardiac marker detection [20]. Troponin, CK-MB, and myoglobin are traditional myocardial markers of myocardial injury, whereas BNP reflects the ventricular function, and thus, is a predictor of impaired ventricular function [21, 22]. In 2008, a consensus of experts on BNP affirmed the application value of blood BNP detection in the adult cardiovascular system [12]. In a study of heart failure in children, Auerbach et al. [9] found that an elevated BNP level is an independent risk factor for prognosis in children with heart failure.

Because severe HFMD is often accompanied by obvious cardiopulmonary dysfunction, we examined the clinical value of the serum BNP level in the diagnosis and prognosis of HFMD. Our results show that the mean serum BNP level in 24 critical HFMD patients was sharply increased to an median concentration of 38.83 pmol/L, with the peak volume even reaching as high as 343.99 pmol/L. This index was significantly higher in the critical HFMD group than in the other groups (*p*˂0.01). However, the serum BNP levels did not differ significantly among the severe HFMD, common HFMD, and control groups. The mechanism of the BNP elevation in critical HFMD is not yet clear. A possible mechanism could be abnormal sympathetic hyperfunction in children with critical HFMD, as such hyperfunction stimulates catecholamine release into the blood, causing an increase in blood flow to the heart to increase ventricular load [6, 13, 14]. Meanwhile, increased resistance in the peripheral vasculature results in increased ventricular afterload to trigger BNP secretion. In addition, under the stress of viral toxins, some inflammatory cytokines can be released, resulting in myocardial damage to induce the release of BNP. These comprehensive factors may result in critical HFMD in pediatric patients with an abnormally increased plasma BNP level. Next, we compared the indexes between patient groups with complications and without complications as well as between survivors and non-survivors group and found that BNP is a risk factor for complications and death in HFMD patients. Its sensitivity and specificity for detecting HFMD were higher. The corresponding optimal threshold values were 8.4 pmol/L for predicting complications and 41.1 pmol/L for predicting death. However, as in common HFMD, BNP alone has a lower specificity. Because BNP is an indicator of heart ventricle injury, for use in the diagnosis of HFMD, it should be considered in combination with other HFMD symptoms and Glu and PaO_2_/FiO_2_ data.

WBC and Glu were found to early warning indicators of severe HFMD [5]. Overall, 22 of 24 HFMD patients (91.7%) with complications had a Glu level greater than 6.1 mmol/L, and the Glu levels in the other two cases (8.3%) were 3.9–6.1 mmol/L. No significant differences were observed between the other three indexes. Overall, our results show that a Glu level of 6.1 mmol/L could be an indicator for complications in HFMD. Lac levels were significantly greater in the critical HFMD group compared with levels in other groups, which reflects the presence of hypoxic injury. Similarly, the patient groups that experienced complications or death presented Glu levels higher than those of patients without complications and survivor. This hypoxic injury could be caused by cardiopulmonary dysfunction and failure.

The oxygenation index is important for monitoring clinical respiratory function, and was assessed in terms of the PaO_2_/FiO_2_ ratio among the different groups of HFMD patients in this study. The normal level is around 400–500 mmHg. A PaO_2_/FiO_2_ less than 300 mmHg is suggestive of respiratory dysfunction. Our results showed that the PaO_2_/FiO_2_ levels were significantly decreased in the critical HFMD group, the group with complications, and the group that died, indicating a dysfunctional state of the respiratory system. However, since mechanical ventilation therapy is an ordinary measure for severe HFMD patients clinically, the exact level of mechanical ventilation in patients with severe HFMD should be carefully planned and controlled. In addition, our results suggest that this index could not be used as an indicator of severe HFMD due to the possible interference of artificial ventilation.

## Conclusion

In this study, we enrolled 83 HFMD patients with or without severe complications treated in our hospital from April 2014 to March 2015. The serum BNP level and other common clinical biochemical indexes were examined and analyzed according to the severity of HFMD (common, severe, and critical; with and without complication; and survivors and non-survivors). The results indicated that the serum BNP level could be a useful biochemical indicator for severe (critical) disease, complications, and death among HFMD patients. When used in combination with Glu and PaO_2_/FiO_2_ measurements as well as HFMD clinical symptoms, the new indicator (BNP) becomes more precise and specific. Our results may provide another valuable biochemical indicator for the clinical diagnosis and prognosis of severe HFMD.

## Abbreviations

BNP: B type natriuretic peptide
CA16: Coxsackievirus A16
CO: Cardiac output
EV71: Enterovirus 71
HFMD: Hand-foot-mouth disease
PaO_2_: Arterial partial pressure of oxygen
